# Primary Open-Angle Glaucoma in Individuals of African Descent: A Review of Risk Factors

**DOI:** 10.4172/2155-9570.1000450

**Published:** 2015-07-31

**Authors:** Rebecca Salowe, Julia Salinas, Neil H Farbman, Aishat Mohammed, Joshua Z Warren, Allison Rhodes, Alexander Brucker, Meredith Regina, Eydie Miller-Ellis, Prithvi S Sankar, Amanda Lehman, Joan M O’Brien

**Affiliations:** Scheie Eye Institute, University of Pennsylvania, Philadelphia, PA, USA

**Keywords:** Glaucoma, Primary open-angle glaucoma, Blacks, African, Risk factors, Blindness

## Abstract

**Objective:**

To identify the major risk factors for primary open-angle glaucoma (POAG) in individuals of African descent.

**Methods:**

We searched PubMed for relevant articles, with results spanning April 1947 to present. All abstracts were reviewed and, where relevant to POAG and race, articles were catalogued and analyzed. Additional sources were identified through citations in articles returned by our search.

**Results:**

Numerous potential POAG risk factors were identified and organized into categories by demographics (age, sex, and skin color), lifestyle choices (smoking, alcohol), comorbidities (hypertension, diabetes, and obesity), ophthalmic findings (eye structure, central corneal thickness, corneal hysteresis, elevated intraocular pressure, myopia, cataract, and vascular abnormalities), family history, socioeconomic status, and adherence. Older age, male sex, lower central corneal thickness, decreased corneal hysteresis, elevated intraocular pressure, myopia, vascular abnormalities, and positive family history were definitively associated with increased risk of POAG.

**Conclusions:**

Individuals at greatest risk for POAG should be screened by an ophthalmologist to allow earlier detection and to slow disease progression. Further studies on the genetics of the disease will provide more insight into underlying pathologic mechanisms and could lead to improved therapeutic interventions. Continued research in urban areas with large populations of blacks is especially needed.

## Introduction

Glaucoma is the leading cause of irreversible blindness worldwide, affecting approximately 70 million people [1]. Primary open-angle glaucoma (POAG), the most common form of the disease, develops as retinal ganglion cell damage causing optic nerve degeneration with subsequent progressive, irreversible vision loss [2]. Individuals of African descent are disproportionately affected by POAG. POAG develops earlier [3–8], presents with greater severity [3–5,9–12], and progresses more rapidly [3–8,13–15] in these individuals. Blacks with POAG also reach adverse endpoints more frequently, including worse visual fields and optic disc cupping [16–20], blindness [6,21,22], vision-related decrease in quality of life [23–28], and increased mortality [29,30].

Poor understanding of the etiology of POAG has hindered attempts at early identification and treatment of this disease. All studies agree that POAG is a complex and multivariate disease. As of now, elevated intraocular pressure (IOP) remains the only treatable component of this disease, but high IOP is neither necessary nor sufficient to develop glaucoma [31]. In order to improve prevention and treatment, it is important to understand the many other risk factors associated with POAG and their relationships to each other. Several factors have been definitively linked to POAG, but the mechanisms of their association to POAG remain inconclusive.

The purpose of this review is to provide a comprehensive analysis of the major risk factors associated with POAG in individuals of African descent. We carefully reviewed all available manuscripts from April 1947 to the present to complete this review. Identifying POAG risk factors in black populations will improve glaucoma screening by allowing clinicians to identify individuals at greatest risk who warrant closer monitoring. Furthermore, by providing an in-depth analysis of each risk factor, we hope to lay the foundation for more focused and personalized treatment of POAG. Finally, comprehensive understanding of POAG risk factors will inform data collection for large prospective studies of this population, including the Primary Open-Angle African-American Glaucoma Genetics study (POAAGG).

## Methods

We searched PubMed using the following terms: glaucoma and (race or ethnic or afro or Africa or black or negro). Dates were unrestricted, and our results spanned April 1947 to the present. All abstracts were reviewed and, where relevant to POAG and race, articles were catalogued and analyzed. Additional sources were identified through citations in articles returned by our search. We restricted our search to English language articles.

## Results

### Demographics

#### Age

POAG risk significantly increases with age in all populations, including blacks [32–34]. The (POAAGG) study found that African-American patients over age 80 were five times more likely to develop POAG than those aged 50–59 (33.0% versus 9.2%, p<0.001, sex-adjusted) [35]. Older black populations also have a tendency to present with more advanced disease at diagnosis, including severe optic nerve cupping or extensive visual field loss [18,36]. In addition, older untreated glaucoma patients and suspects experience more rapid disease progression than their younger counterparts [20].

#### Sex

Most reports suggest that black males have a greater risk of POAG than black females. A retrospective Barbados study and POAAGG both reported significantly greater POAG risk in males after adjusting for age (Barbados: 1.66 [1.24–2.24]; POAAGG: (1.29 [1.20, 1.40]) [34,35]. Studies examining African Caribbeans [32], Nigerians [37], and Congolese [38,39] found no significant associations between sex and POAG, but each trended toward greater risk in males.

The trend towards greater POAG risk in males may be stronger than it appears. Women account for 60% of eye care related outpatient visits, with older women more than twice as likely to have an appointment as older men [40]. Thus, males may be underdiagnosed with POAG and have even higher rates of POAG than studies indicate.

#### Skin pigmentation

While there is no definitive association between skin pigmentation and POAG, there is a trend between darker skin pigmentation and higher IOP among blacks. Wormald et al. suggested that darker skin may be associated with elevated IOP but not POAG [32], a result subsequently confirmed in Barbados [41]. African-Americans in Cleveland showed no relationship between skin color and IOP, but this study examined a much smaller cohort (n=213) [42] than the previous two studies (n=873 and n=4631, respectively) [32,41].

### Lifestyle choices

#### Smoking

There is no definitive association between cigarette smoking and POAG. Several studies have shown a positive association between smoking and POAG [38,43], while others failed to confirm these findings [32,44,45]. However, these studies focused on very small cohorts with unmatched case-control groups [32,38,43–45] or examined only the short-term effects of smoking [43]. More large-scale studies examining the long-term effects of smoking on POAG risk are needed to confirm these findings. It is important to note that these results may be complicated by patient self-reporting or by IOP increases from nicotine [43].

#### Alcohol

Alcohol consumption has not previously been associated with POAG. The Baltimore Eye Survey [44] and a Congolese study [39] found no association between alcohol usage and POAG. Age-adjusted history of alcohol use was also not associated with glaucoma in the POAAGG study [46]. A prospective cohort of African-American women showed significantly greater POAG risk among current alcohol users versus never-users, but these results may be biased, since included subjects were more likely to report alcohol consumption than excluded subjects [45].

### Comorbidities

#### Hypertension

A clear and direct relationship between blood pressure (BP) and POAG has not been established in black populations. The majority of studies found no significant relationship between BP and POAG [32,34,38,39,44,47]. Several other studies have assessed ocular perfusion with arterial blood pressure measurements, as abnormal ocular perfusion has been associated with optic nerve damage [33]. The Barbados Eye Study found an association between lower systolic, diastolic, and mean ocular perfusion pressure and POAG [33]. Conversely, two Nigerian studies found higher systemic BP and ocular perfusion pressure significantly associated with POAG [37,48]. The relationship between ocular pressure measurements and arterial blood pressure requires further investigation.

#### Diabetes

Most reliable studies found no relationship between diabetes mellitus (DM) and POAG [33,34,38,49], while a few demonstrated a protective relationship [50,51]. It is possible that this potential protective factor results from DM patients being more likely to have annual eye exams, which allows for earlier detection of POAG. Several studies have reported positive relationships between DM and POAG [32,37,48], but these studies were not age-adjusted [32,48], used unstandardized criteria for POAG diagnosis [37], or included unclear methodology for diabetes diagnosis [48].

#### Obesity

Studies have found both positive and inverse relationships between body-mass index (BMI) and POAG. The Barbados Eye Study found that elevated BMI was protective against POAG after adjusting for hypertension and diabetes [39], a result subsequently confirmed by the POAAGG study [46]. However, a Congolese case/control study found increased BMI significantly associated with POAG [38], while two other studies did not find a positive relationship between high BMI and POAG [34,39]. Standard BMI measurements may not account for the genetic mechanisms influencing body mass, making this variable difficult to accurately measure and correlate with POAG prevalence [39]. New, more targeted body mass and adiposity measurement tools could also prove helpful in elucidating whether adiposity in certain body regions is correlated with POAG.

### Ophthalmic findings

#### Eye structure

Several studies in black populations have identified ocular structural features correlating with POAG [33,52–54]. However, because glaucoma affects eye structure, it is difficult to determine whether these findings represent risk factors for glaucoma development or effects of glaucoma pathology. Studies demonstrating eye structure differences between healthy blacks and whites could better represent true POAG risk factors and help explain the higher prevalence of POAG in blacks [55]. In particular, multiple studies have demonstrated that healthy blacks have thinner central corneal thickness (CCT) [55–62] and larger optic discs [15,56,58,63–71] than their Caucasian counterparts. Prospective studies would be necessary to determine if these structural differences correlate with higher POAG risk.

#### Central corneal thickness (CCT)

As discussed above, healthy blacks have a thinner CCT than healthy whites [53,55,66,68,69,72–74]. Several studies have shown that CCT affects IOP readings [72]. However, the Goldmann applanation technique, considered the gold standard for measuring IOP, is based on the assumption that corneal thickness does not vary significantly between individuals. This assumption may lead to underestimation and possible undertreatment of blacks’ IOP and could lead to increased optic nerve damage in this population [55–62].

#### Corneal hysteresis

Recent studies have shown that corneal hysteresis (CH), an indicator for the viscoelastic properties of the cornea, is lower in healthy Africans than healthy Caucasians [73,74]. The increased elasticity of black eyes may cause underestimation of IOP [72]. In addition, having lower hysteresis increases the risk of visual field progression [75,76] and optic nerve damage [77]. In recent retrospective studies, low CH was a stronger predictor for progression than thin CCT, although it may be more of an association rather than a clear risk factor [74,77].

#### Elevated IOP

Elevated IOP has been confirmed by many studies as a strong risk factor for POAG. The Baltimore Eye Survey found that the risk of optic nerve damage increased with IOP [31], a finding replicated by the Barbados Eye Study [34]. The Barbados Eye Study showed POAG risk increased 12% for each 1 mmHg increase in IOP [78] and that IOP>21 mmHg imparted 11-fold greater odds of POAG [34].

Additionally, IOP can fluctuate naturally throughout the day [79,80]. Some studies have even suggested that this fluctuation may be a more important risk factor for POAG than a single baseline measurement [81].

While elevated IOP is a significant risk factor for POAG, it is neither necessary nor sufficient to develop glaucoma. Many POAG subjects have normal baseline IOP, as indicated in several studies [14,78,82–84].

#### Myopia

Most studies have found a correlation between myopia and POAG [37,85–88], although this relationship is not fully understood. Some hypothesize that severe myopia changes the connective and nerve tissue architecture, which could structurally alter the optic nerve head and make it more susceptible to glaucomatous damage [89]. Myopic eyes also have reduced retinal nerve fiber layer thickness, which could be a risk factor for developing POAG [89]. However, the Barbados Eye Study found that POAG patients developed subsequent myopia [82]. Myopia and POAG could share some common mechanistic pathways since both involve changes in ocular connective tissues and nerve fiber layer, so it is difficult to ascertain which condition predisposes to the other.

#### Cataract

Cataracts and POAG are common co-morbidities, as the conditions are the first and second leading causes of blindness worldwide and are prevalent in the aging population. However, a definitive correlation between these co-morbidities has not been established. The Barbados Eye Study found that cataracts were more common among POAG cases than controls, which could be due to ascertainment bias [34]. Several other studies have failed to confirm a cataract/POAG association [32,33].

#### Vascular abnormalities

Persons of African descent tend to have more systemic vasculature complications than whites. In 2010, the rate of death per 100,000 attributable to cardiovascular disease (CVD) was 278.4 for white males, 369.2 for black males, 192.2 for white females, and 260.5 for black females [90]. Blacks also have higher rates of diabetes and hypertension [8,50,63]. Consistent with these findings, persons of African descent have significantly lower blood flow in all retrobulbar blood vessels than whites [91]. According to the vascular theory of glaucoma, insufficient blood flow to the optic nerve can cause neuropathy and may be a risk factor for the more severe glaucoma observed in blacks [92].

### Family history

POAG risk strongly correlates with family history in black populations, reflecting heritability and/or shared environmental factors [49]. Studies in Nigeria [37], Barbados [33,34], Congo [38], and Baltimore [49] demonstrate odds of POAG up to 18-fold higher with positive family history [38]. Siblings of an affected patient were at the highest risk of developing POAG compared to parents or children [49]. This finding was subsequently confirmed by the Barbados Eye Study, which found a 4.5 odds ratio of POAG patients having a positive sibling history [38]. Maternal family history was reported twice as often as paternal history in POAG patients [38], a result consistent with the theory that mitochondrial DNA may be involved in the pathogenesis of POAG [93–95].

Evaluating family history can introduce many biases. Incomplete information about family members’ health, tendency to know maternal lineage better than paternal lineage, and lack of familiarity with glaucoma as a diagnosis make this variable difficult to measure reliably [32]. In the Baltimore Eye Survey, previously diagnosed individuals reported POAG family history significantly more often than newly diagnosed individuals, suggesting that individuals are less aware of their family history upon initial diagnosis [49].

### Socioeconomic status

Socioeconomic deprivation is associated with late diagnosis [96] and more severe POAG at presentation [97,98]. Socioeconomically deprived groups also have demonstrated a higher need for information on the practical aspects of POAG, such as ocular medication usage and social support for visually-impaired individuals. These groups are also less likely to understand the irreversibility of POAG damage [99] and are less aware of glaucoma in the family [98].

These findings particularly affect those of African descent, who tend to have a lower average socioeconomic status than other ethnic groups. In 2010, 23% of black adults lived below the poverty level, compared with 11% of Asian and 9% of non-Hispanic white adults [100]. Census data from the same year indicates that only 19.8% of blacks in the US aged 25 years and older were college graduates compared with 30.3% whites and 52.4% Asian and Pacific Islander [101].

While lower socioeconomic status may create barriers for blacks that lead to later diagnosis and more severe disease, POAAGG recently reported that poverty does not increase risk of a POAG diagnosis in African-Americans [35]. This suggests that lower socioeconomic status may contribute to worse initial disease, but it is not the predominant cause of the higher prevalence of POAG in blacks.

### Adherence

Since IOP remains the only treatable factor in glaucoma, adherence is essential to maintain a lower IOP and to slow glaucomatous optic nerve damage. Previous studies have shown that black patients have lower adherence rates than other races, particularly males, independent of education and socioeconomic status [102]. According to one study, the top five barriers to adherence were forgetfulness, drug side effects, cost, difficulties with ocular medication administration, and eye drop schedules [103].

However, adherence is a complex issue to understand and to study across races. Black patients, for example, are prescribed more eye-drops than their white counterparts [104], increasing the likelihood that eye drop schedules and cost hinder adherence. In addition, poor health literacy has been significantly correlated with non-compliance and poor understanding of glaucoma [105,106]. In one study, 56% of African-Americans had poor health literacy compared to 16% of Caucasians [100]. Since POAG is asymptomatic until vision loss, those with poor health literacy may be less likely to adhere to medication if they do not fully understand how it will benefit them. Poor health literacy and lower socioeconomic status may also contribute to African-American patients choosing other necessities over ocular medications. Improving patient adherence to their medication and appointment schedule may help to control IOP and to slow further glaucomatous damage.

## Conclusion

This review provided a comprehensive analysis of the major risk factors associated with POAG in individuals of African descent. By examining all relevant research since April 1947, we found that older age, male gender, lower CCT, decreased CH, elevated IOP, myopia, vascular abnormalities, and positive family history were significantly associated with POAG in individuals of African descent. Individuals at greatest risk should be screened earlier and more frequently for POAG.

In addition to screening those at greatest risk and implementing protective measures to slow POAG progression, we also recommend future studies to concentrate on the genetic component of POAG, which remains understudied. Determining the function of associated genetic variants will provide insight into the greater risk for glaucoma development and progression in individuals of African descent and pave the way for improved and more targeted therapeutics.
